# Gene Expression Profiles in Rice Developing Ovules Provided Evidence for the Role of Sporophytic Tissue in Female Gametophyte Development

**DOI:** 10.1371/journal.pone.0141613

**Published:** 2015-10-27

**Authors:** Ya Wu, Liyu Yang, Aqin Cao, Jianbo Wang

**Affiliations:** State Key Laboratory of Hybrid Rice, College of Life Sciences, Wuhan University, Wuhan, 430072, China; Institute of Crop Sciences, CHINA

## Abstract

The development of ovule in rice (*Oryza sativa*) is vital during its life cycle. To gain more understanding of the molecular events associated with the ovule development, we used RNA sequencing approach to perform transcriptome-profiling analysis of the leaf and ovules at four developmental stages. In total, 25,401, 23,343, 23,647 and 23,806 genes were identified from the four developmental stages of the ovule, respectively. We identified a number of differently expressed genes (DEGs) from three adjacent stage comparisons, which may play crucial roles in ovule development. The DEGs were then conducted functional annotations and Kyoto encyclopedia of genes and genomes (KEGG) pathway analyses. Genes related to cellular component biogenesis, membrane-bounded organelles and reproductive regulation were identified to be highly expressed during the ovule development. Different expression levels of auxin-related and cytokinin-related genes were also identified at various stages, providing evidence for the role of sporophytic ovule tissue in female gametophyte development from the aspect of gene expression. Generally, an overall transcriptome analysis for rice ovule development has been conducted. These results increased our knowledge of the complex molecular and cellular events that occur during the development of rice ovule and provided foundation for further studies on rice ovule development.

## Introduction

Angiosperm plants undergo two phases in their life cycle: the haploid phase of male and female gametophytes and the diploid sporophyte phase. Not only male and female gametogenesis but also the fertilization of haploid gametophytes takes place surrounded by the diploid sporophyte tissues. The ovule is the site of megasporegenesis and fertilization in angiosperm, and is the major component of the female reproductive organ. The role of the ovule in sexual reproduction is critical.

Early studies have provided insight into the development of rice ovules in terms of morphological and cellular features [1–3]. The development of rice ovule is initiated from the ovule primordium. Consisting of a round mass of actively dividing cells, the main portion of the primordium becomes the nucellus. Below the nucellus epidermis, a large cell develops into the archespore. Subsequently, it enlarges and differentiates into the megaspore mother cell (MMC) [4]. After the meiotic division of the MMC, a linear tetrad of megaspores is produced. Three megaspores at the micropylar pole soon degenerate, leaving the chalazal megaspore as the functional megaspore. The surviving functional megaspore then undergoes three rounds of mitosis, resulting in the formation of an eight-nucleate female gametophyte, followed immediately by nuclear migration and cellularization. Finally, the embryo sac matures with the polarization and differentiation of the egg cell and two synergids at the micropylar end, three antipodal cells at the chalazal end and two polar nuclei in the central cell. In the process of fertilization, the egg cell and the central cell fuse with two sperm cells, followed by the formation of zygote and primary endosperm nucleus, while the sporophytic ovule tissue undergoes a programmed cell death (PCD)-triggered degradation after fertilization [5].

However, researches focusing on the development of female gametophyte in rice are limited for several reasons. First, the female gametophyte is deeply embedded in the sporophytic ovule tissue, thus it is difficult to separate it from the surrounding tissue. Although a separation technique that may facilitate research in this field has been established [6], difficulties remain due to the low cellular activity of the separated cells and the low success rate in separation.

Due to the inaccessibility of the relevant cells, ovule-defective mutants facilitate investigation of the molecular mechanism of ovule development. In *Arabidopsis*, genes encoding an extensive range of transcription factor, protein kinase, putative corepressor, mitochondrial ribosomal subunit and a component of the RNA interference pathway have been identified as indispensable for ovule morphogenesis and numerous studies on ovule mutants have emphasized their importance [7]. Based on the various mutation phenotypes, the ovule mutants can be assigned into two classes, sporophytic mutants and gametophytic mutants. The ovule sporophytic mutants represent the mutation that occurred in the sporophytic tissue of the ovule. For instance, the *ino* mutant does not develop the outer ovule integument, but the inner integument is normal. Synchronously, the embryo sac is gametophytically defective, indicating the important role of the sporophytic ovule tissue in promoting female gametophyte development [8]. A frame-shift of the Bs-group MADS-box gene causes defective development of the integument [9]. Furthermore, down-regulation of OsIG1 will lead to the formation of unusual double ovules and abnormal female gametophyte [10]. The ovule gametophytic mutations are identified as a distorted segregation ratio or reduced seed set because they fail to transmit through the egg cell. For example, the *ig1* (indeterminate gametophyte 1) mutant in maize has a prolonged phase of free nuclear divisions leading to a variety of embryo sac abnormalities (including extra polar nuclei, synergids and egg cells) [11]. The ethylene-response *ctr1* (constitutive triple response 1) mutant resulted in a defective embryo sac leading to distorted segregation [12]. The *CTR1*gene encodes Raf-like Ser/Thr protein kinase, which is the key enzyme for ethylene signal transduction [13, 14]. This gene is involved in cell-cell communication between the sporophyte and female gametophyte [15, 16]. Based on the genetic evidence of studies on ovule-defective mutants, the sporophytic maternal tissues are hypothesized to dominate female gametophyte formation [17, 18]. Moreover, comparative transcriptome analysis of *sporocyteless* (*spl*) and *coatlique* (*coa*) ovules has indicated a response of the embryo sac to sporophyte ovule tissue and mutual communication between sporophytic ovule tissue and the embryo sac [19].

Although research on mutants can promote an understanding of the functions of particular genes in the context of biological phenomena, these approaches have failed to provide more detailed information regarding other components of the regulatory circuitry positioned upstream or downstream in the hierarchy. However, transcriptome analysis based on microarrays can provide more detailed genetic information. Using this technique, dynamic gene expression was shown in the developing rice ovule (*Oryza sativa* L.ssp *japonica* cv. Nipponbare) [20], establishing a new insight into the research of female development. Because the transcriptome data were obtained using microarray-based methods, inherent limitations such as providing only a fragmented view of transcriptome patterns still exist. Rather than measuring relative gene expression, the RNA sequencing (RNA-seq) generates absolute information; thus, many inherent limitations of microarray analysis can be avoided. This technique has been used to study the developmental process of the embryo and endosperm in rice [21, 22]. Transcriptome analysis of developing ovule is needed to understand female development in rice. However, current transcriptome information regarding the rice ovule is incomplete and many genes have not been identified, hindering the analysis of genetic and molecular mechanisms involved in ovule development.

To obtain greater insight into the genetic and molecular processes underlying ovule development in rice, we used RNA-seq technology to analyze the transcriptome of ovules at four developmental stages (embryo sac mother cell during meiosis, megaspore mitosis, mature embryo sac and two days after flowering). Using deep RNA-seq (Illumina RNA-seq method), sufficient and accurate transcriptome data were generated. We then conducted Gene Ontology (GO), KEGG, MapMan pathway, hierarchical clustering and transcription factor expression analyses, which are more comprehensive and systematic than the methods utilized in similar studies. In this study, we discovered transitions in gene expression profiles of developing rice ovules, which can provide genetic and molecular evidence of the cell-cell communication between sporophytic ovule tissue and female gametophyte during the ovule developmental process. This is the first report on the application of RNA-seq to transcriptome analysis of developing ovules in rice. Furthermore, our research will serve as a database for the transcriptome of developing ovule in this field.

## Materials and Methods

### Plant materials

The rice (*O*. *sativa* L. ssp. *indica* cv. 9311) plants were grown and maintained in the greenhouse of Wuhan University (China).

The development of ovule before fertilization was dissected into three stages from OVR1 to OVR3: OVR1 represented ovule containing MMC process; OVR2 was defined as ovule at megaspore mitosis; OVR3 represented ovule containing mature embryo sac. OVR4 represented the ovule of two days after fertilization, which was designed to study the effect of fertilization on ovule gene expression. In order to compare the different gene expression between the female reproductive organ and vegetative organ, transcriptomic analysis of the rice leaf was also conducted. The leaf used in this study was boot leaf, which was represented with LEAF, and it was collected for three biological replicates before grinded in liquid nitrogen for later use.

According to the correspondence existed between the morphological features of rice floret and female gametophyte development, the sampling stage of ovule was determined based on the anther length [20]. In order to make sure of the sampling stage of the ovule, the morphological features inside the ovule of each developmental stage were observed using the method of DIC (differential interference contrast) [23]. All ovules in our work were taken from ovaries using micro dissection needles under an integrated microscope and stored in liquid nitrogen for mRNA extraction. For each developmental stage, three biological replicates were performed. Each replicate was collect from more than 150 florets of the 20 different plants randomly selected during the growing season.

### Preparation of cDNA libraries and RNA sequencing

The RNA was extracted utilizing Trizol reagent according to the manufacturer’s instruction (Invitrogen, Canada). Absorbance at 260 and 280 nm was used to determine the yield and purity of RNA samples. RNA 6000 Nano Assay Kit (Agilent, USA) and Agilent 2100 Bioanalyzer (Agilent, USA) were applied to check the RNA integrity. For the procedures above, three biological replicates of each sample were conducted individually. Before the preparation of library, equal amounts of the RNA from three replicates were pooled together. Then, the RNA samples were used to construct the cDNA libraries by Illumina’kit (Illumina, USA). The libraries were finally sequenced via Illumina HiSeq^TM^ 2000.

### Mapping reads to the reference genome

The reads generated from RNA sequencing were mapped to the reference genome from rice (http://rice.plantbiology.msu.edu) as that described by Xu [21].

### Normalization of the genes expression from RNA-seq

ERANGE software (version 4.0) was used to evaluate gene expression by assigning reads to their site of origin and to count them (http://woldlab.caltech.edu/gitweb/). Raw digital gene expression counts were normalized by a variation of the reads per kilobase per million mapped reads (RPKM) method. The calculated values could be directly used for the comparison of the different gene expression among samples.

### Identification of DEGs

The R package DEGseq was applied to identify DEGs with the random sampling model based on the read count for each gene at different developmental stages [24]. In multiple test and analysis, the false discover rate (FDR) was used to determine the threshold of *P* value. We used FDR≤0.01 and the absolute value of log_2_Ratio≥1 as the threshold for judging the significance of each gene expression difference.

### GO, KEGG pathway and MapMan analysis

GO annotations (http://www.blast2go.org/) were performed with the Blast2GO program (version 2.3.5) for the expressed genes. Then the GO functional classifications of genes were done by web gene ontology annotation plot (WEGO) (http://wego.genomics.org.cn/) with a robust FDR correction to get an adjusted *P*-value.

KEGG pathway analysis were performed using Cytoscape software (version 2.6.2) (http://www.cytoscape.org/) with the ClueGO plugin (http://www.ici.upmc.fr/cluego/cluegoDownload.shtml).

The MapMan tool (http://MapMan.gabipd.org) was used to visualize the involvement of the expressed genes in the pathways, and the method was described as Gao [22].

### Real-time quantitative PCR validation of RNA-seq data

To validate the data of RNA-seq, nineteen genes were chosen randomly for quantitative real-time polymerase chain reaction (qRT-PCR) analysis. Specific primers for qRT-PCR were designed with the software of Primer (version 5.0). *OsActin1* (LOC_Os03g50885) were used as the internal reference gene control [20]. The qRT-PCR was carried out as described by Gao [22]. The analyses of qRT-PCR were performed in triplicate with three biological replicates.

## Results

### Illumina sequencing and alignment to the reference genome

**Fig 1:**
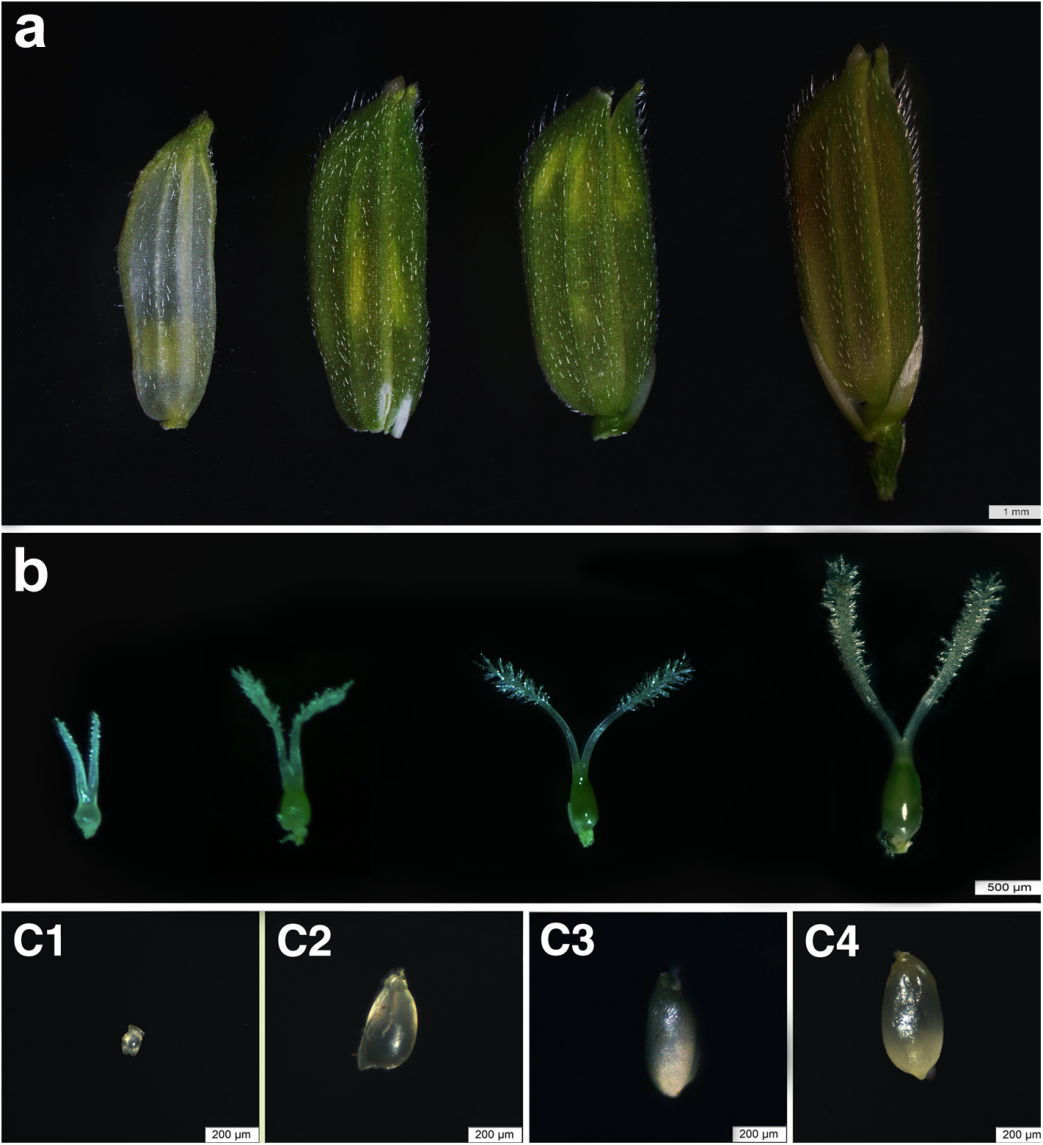
The Morphological observation of the developing florets, pistils and ovules. **A** Florets at three developmental stages. **B** Pistils at three developmental stages. **C1** Ovule in OVR1 stage. **C2** Ovule in OVR2 stage. **C3** Ovule in OVR3 stage. **C4** Ovule in OVR4 stage.

The rice ovules at four stages were collected according to the anther length [20], and they were confirmed with the method of DIC. The DIC images clearly displayed the inner structure of the ovules and showed the consistency to result of the sampling method depending on the anther length, indicating that the sampling stages were correct (Fig 1 and S1 Fig). Thus, the four stages of samples can be used for the following research. In this study, we sequenced five cDNA libraries: OVR1, OVR2, OVR3, OVR4 and LEAF and generated 37,507,626 sequence reads. The number of clean reads for the five samples was 7,826,182(LEAF), 7,433,271(OVR1), 7,044,517(OVR2), 7,586,725(OVR3) and 7,185,948(OVR4) (Table 1). Each library contained more than 7 million reads, indicating that the tag density was sufficient for the quantitative gene expression analysis (S2 Fig). The clean reads were mapped to the rice (*Oryza sativa* L. ssp. *japonica*) genome database (http://rice.plantbiology.msu.edu) using the SOAP aligner/soap2 software, with no more than two-base mismatches in the alignment. Of the total clean reads, 78.59% were uniquely matched on average and were used for gene expression analysis of each library (Table 1).

**Table 1:** Summary of the read numbers mapping to the rice genome based on the RNA-seq data.

	Total reads	Total mapped reads	Unique match	Muti-position match	Unmapped reads
LEAF	7,826,182 (100%)	6,634,796 (84.78%)	6,229,201 (79.59%)	405,595 (5.18%)	1,191,386 (15.22%)
OVR1	7,433,271 (100%)	6,299,385 (84.75%)	5,878,909 (70.09%)	420,476 (5.66%)	1,133,886 (15.25%)
OVR2	7,044,517 (100%)	5,867,534 (83.29%)	5,471,801 (77.67%)	395,733 (5.62%)	1,176,983 (16.71%)
OVR3	7,586,725 (100%)	6,313,602 (83.22%)	5,910,889 (77.91%)	402,713 (5.31%)	1,273,123 (16.78%)
OVR4	7,185,948 (100%)	6,039,335 (84.04%)	5,646,056 (78.57%)	393,279 (5.47%)	1,146,613 (15.96%)

### Genes expressed in developing ovules and leaves

**Fig 2:**
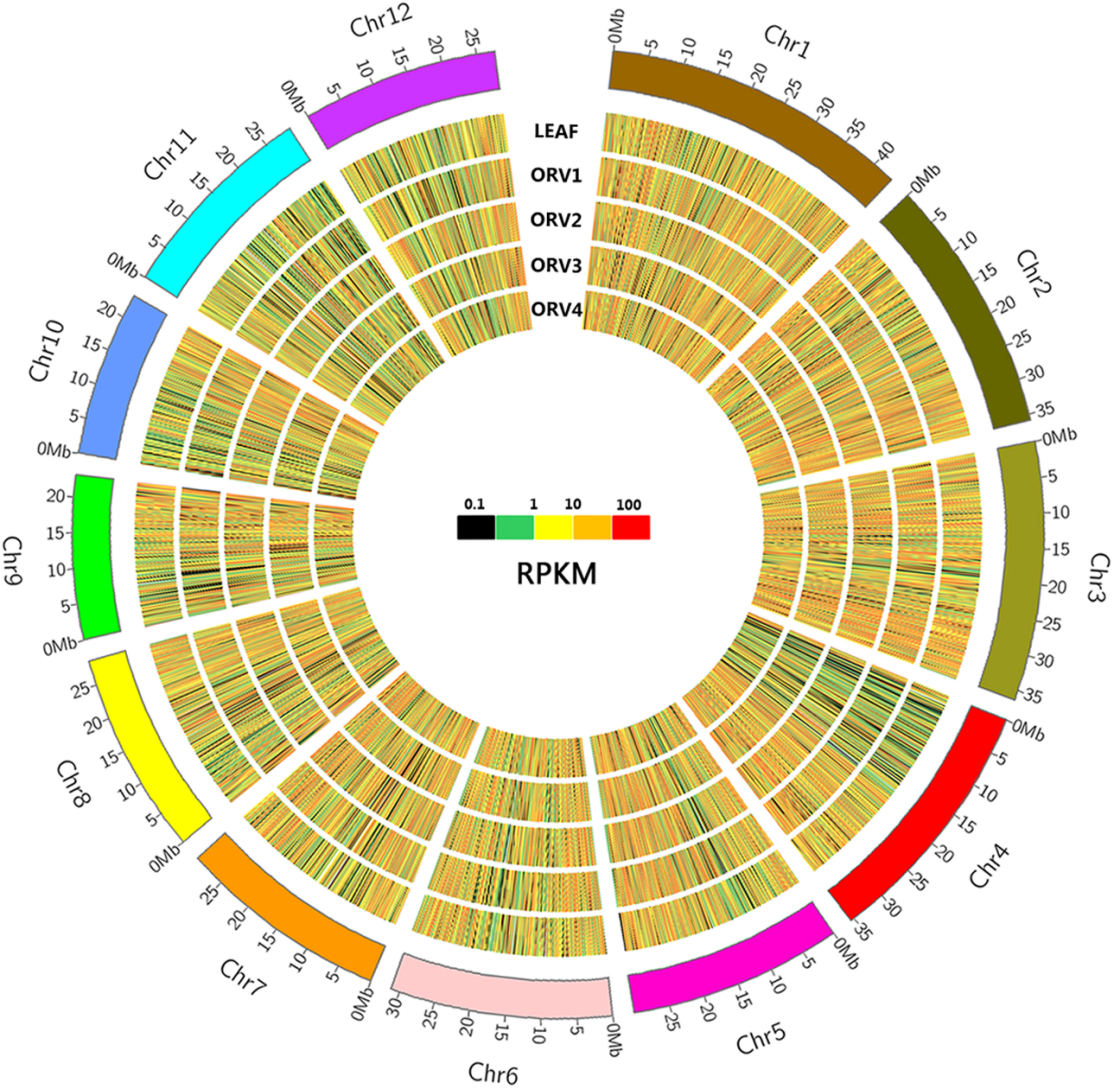
Circos diagram depicting the gene expression in leaf and ovules of four developmental stages. In this figure, the entire rice chromosomes were displayed by the first outer ring. Five concentric rings represented the five samples (LEAF, OVR1, OVR2, OVR3, OVR4), respectively. Colors represented (from black to red) the expression levels of all detected transcripts in every sample based on the RPKM value.

To gain statistical confirmation of gene expression among different samples, the RPKM method was used to normalize the gene expression level. The uniquely matched reads were used to calculate the RPKM value of genes. In total, 30,858 genes were identified in the RNA-seq data, among which 24,920, 25,401, 23,343, 23,647 and 23,806 genes were expressed in LEAF, OVR1, OVR2, OVR3 and OVR4, respectively, providing sufficient data for investigation of ovule development. The size distribution of these identified genes was shown in Table 2. The group of genes with the greatest length (≥ 2,000 bp) comprised the highest proportion of genes (11,054 genes; 35.82% of all genes). The visualization tool Circos facilitates the identification and expression of genes, which is useful for displaying variation in genome structure and any other type of positional relationship between genome intervals [25]. In the present study, the global gene expression profiles of the five samples (LEAF, OVR1, OVR2, OVR3 and OVR4) were visualized using Circos (Fig 2). As shown in Fig 2, the genome-wide analysis of gene expression indicated that the majority of genes were expressed with RPKM values ranging from 0.1 to 100, and that the expressed genes were distributed in the reference genome. We also determined that the genes on Chr4, Chr10, Chr11 and Chr12 were expressed at lower levels, and those on Chr3, Chr5 and Chr7 were expressed at higher levels in all five samples.

**Table 2:** Sequence length distribution of the all 30,858 detected genes in the leaf and four developmental stages of ovules.

Gene length (bp)	Total number	Percentage
100–500	2,099	6.80%
500–1,000	5,264	17.06%
1,000–1,500	6,260	20.29%
1,500–2,000	6,181	20.03%
≥2,000	11,054	35.82%
Total	30,858	100%

**Fig 3:**
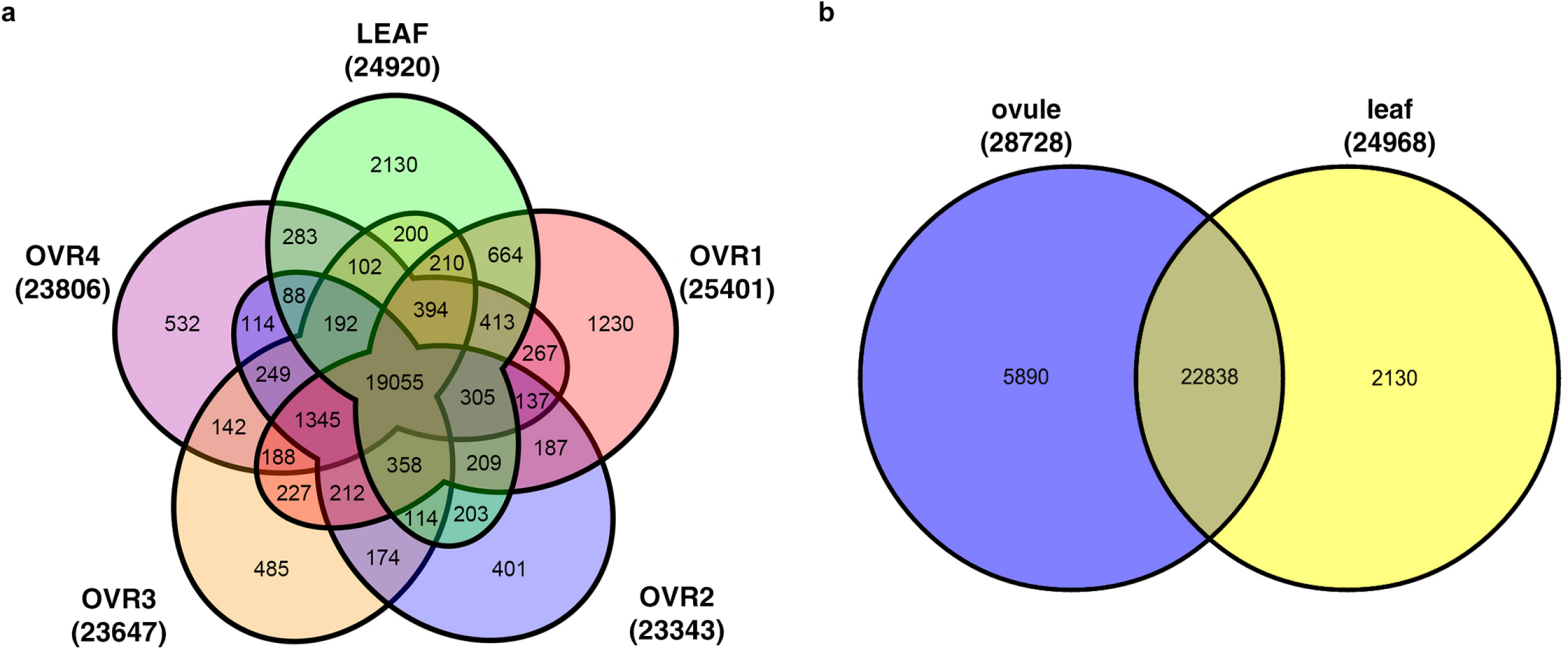
a Venn diagram showing the genes expressed in each of the four stages of rice ovule development and leaf. b Venn diagram showing the genes expressed between the union of four stages of ovule and the leaf.

The distribution of expressed genes in the five samples was visualized using a Venn diagram. The detailed statistical analysis of the identified genes in all five samples was shown in Fig 3A. Among the 30,858 genes, 19,055 (61.75%) genes were expressed in all five samples and they may function as housekeeping genes, while 24,920 genes (80.76%) were expressed in the leaf and 28,728 (93.10%) in the ovule. We compared the genes expressed in the ovules at the four developmental stages. As shown in Fig 3A, the number of genes commonly expressed between the two adjacent ovule stages was 21,808 (OVR1 and OVR2), 21,699 (OVR2 and OVR3) and 21,667 (OVR3 and OVR4), indicating that the number of commonly expressed genes was similar among the ovule developmental stages. When comparing the overall ovule (the union of OVR1, OVR2, OVR3 and OVR4) with LEAF (Fig 3B), 22,838 genes were expressed in both of the ovule and leaf. A total of 5,890 genes were expressed only in the ovule and 2,130 genes were expressed only in the leaf, indicating the gene expression difference between the vegetative organ and the female reproductive organ of rice.

### Analysis of DEGs

**Fig 4:**
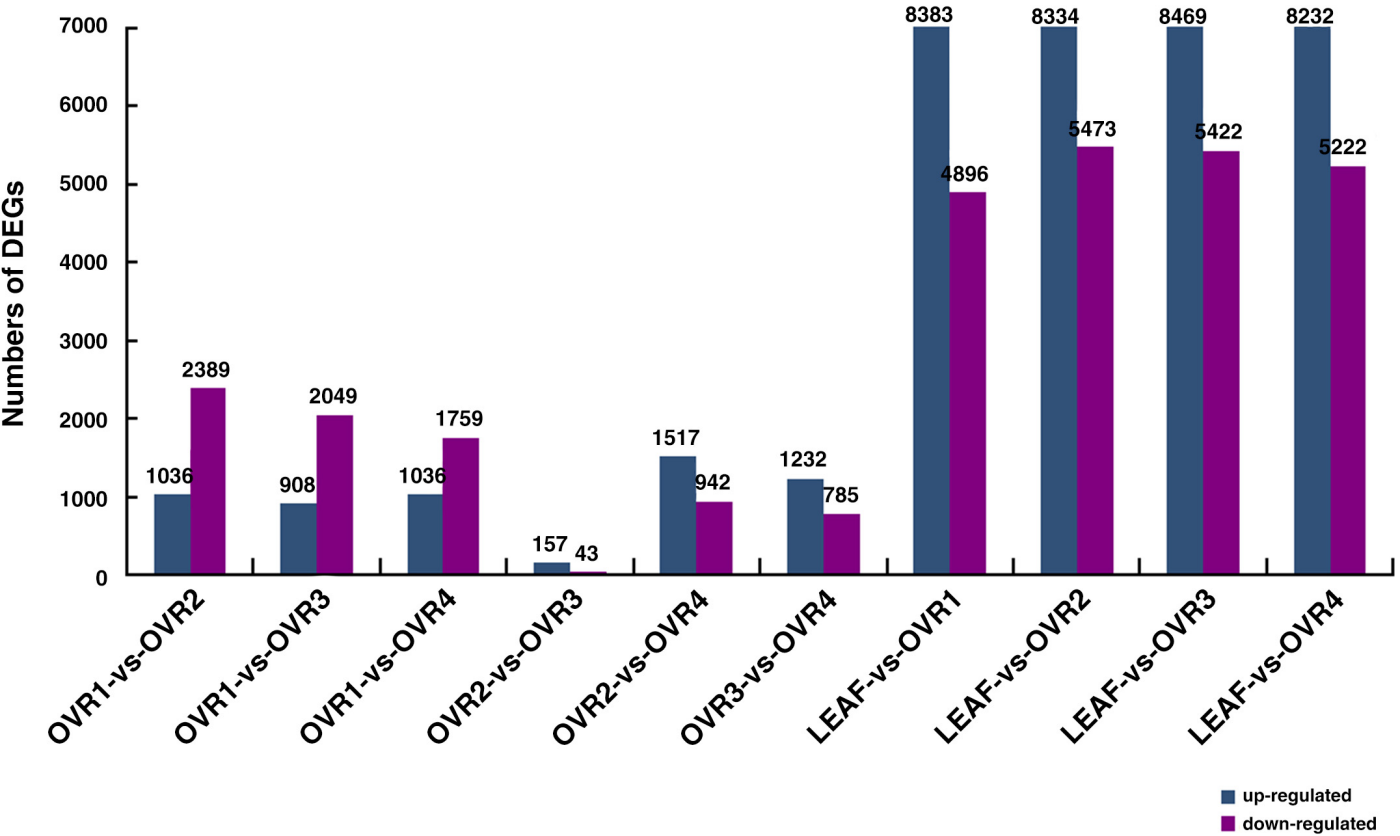
Different expressed genes identified between 10 comparisons (OVR1-vs-OVR2, OVR1-vs-OVR3, OVR1-vs-OVR4, OVR2-vs-OVR3, OVR2-vs-OVR4, OVR3-vs-OVR4, LEAF-vs-OVR1, LEAF-vs-OVR2, LEAF-vs-OVR3, LEAF-vs-OVR4). The numbers of up-regulated and down-regulated genes were revealed.

To identify genes that showed significant changes in expression between each library, DEGs with 10 comparisons (LEAF-vs-OVR1, LEAF-vs-OVR2, LEAF-vs-OVR3, LEAF-vs-OVR4, OVR1-vs-OVR2, OVR1-vs-OVR3, OVR1-vs-OVR4, OVR2-vs-OVR3, OVR2-vs-OVR4 and OVR3-vs-OVR4) were identified by a strict algorithm that stipulates the following: FDR≤0.001 and the absolute value of log_2_Ratio≥1. The statistical analysis of DEGs for each comparison was shown in Fig 4. Greater disparity was found in the comparisons between leaf and ovule than that between neighboring developmental stages of the ovules. For example, the number of DEGs between OVR1 and LEAF was 13,279, which was considerably more than that between OVR1 and OVR2. When comparing OVR1 with OVR2, 3,425 genes were identified as DEGs, of which 1,036 genes were up-regulated and 2,389 down-regulated. Compared with OVR2, the OVR3 stage had 157 up-regulated and 43 down-regulated DEGs. When comparing OVR3 with OVR4, 1,232 DEGs were up-regulated and 785 were down-regulated in OVR4. The expression trend of DEGs during ovule development may reflect the molecular mechanism that controls ovule development.

### GO analysis of all detected genes and DEGs among the ovule developmental stages

**Fig 5:**
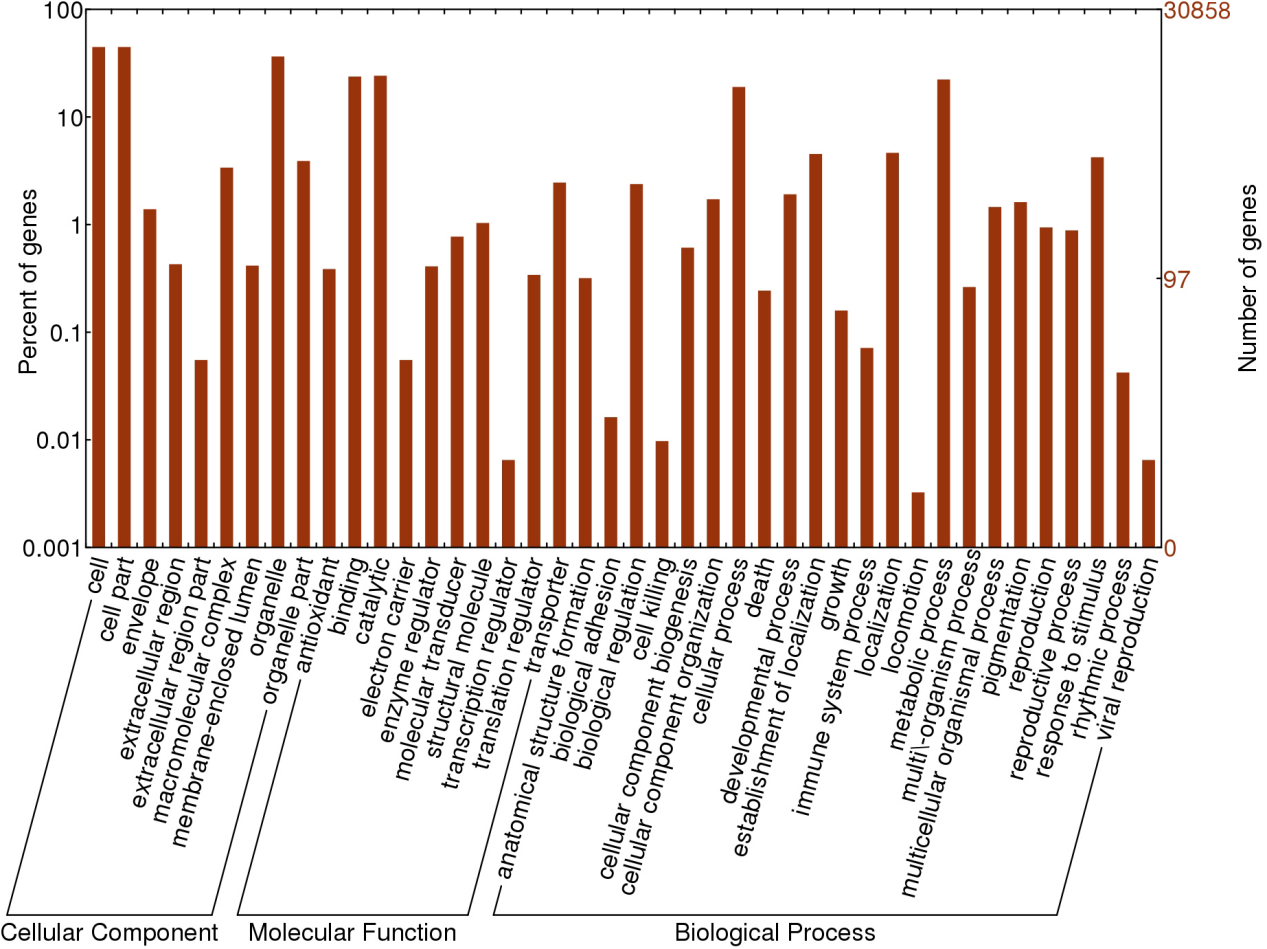
GO classification of all 30,858 genes in leaf and four developmental stages of ovule. The results are summarized in three main GO categories (cellular component, molecular function and biological process. The x-axis represents the name of GO sub-categories. The left y-axis represents the percent of a specific category of genes in that main category. The right y-axis represents the number of genes expressed in the given sub-category.

GO analysis, a common method of classifying gene functions that enables a scientific and straightforward elucidation of identified genes, has three ontologies that describe the biological process, molecular function and cellular component of genes. To facilitate the global analysis of gene expression, all identified genes were mapped to the GO terms in the database (http://www.geneontology.org/) to identify significantly enriched GO terms (Fig 5). Of the 30,858 detected genes, 18,391 (60.0% of the total genes) had at least one gene annotation. According to the secondary classification of GO terms, all annotated genes were classified into 45 functional groups of the three main GO classification categories. Among them, the terms cell (GO: 0005623), cell part (GO: 0044464) and organelle (GO: 0043226) were dominant in the cellular component category. The terms binding (GO: 0005488), catalytic (GO: 0003824) and translation regulator (GO: 0045182) were prominent groups in the molecular function category. The terms cellular process (GO: 0009987), metabolic process (GO: 0008152) and establishment of localization (GO: 0051234) were highly represented in the biological process category.

**Fig 6:**
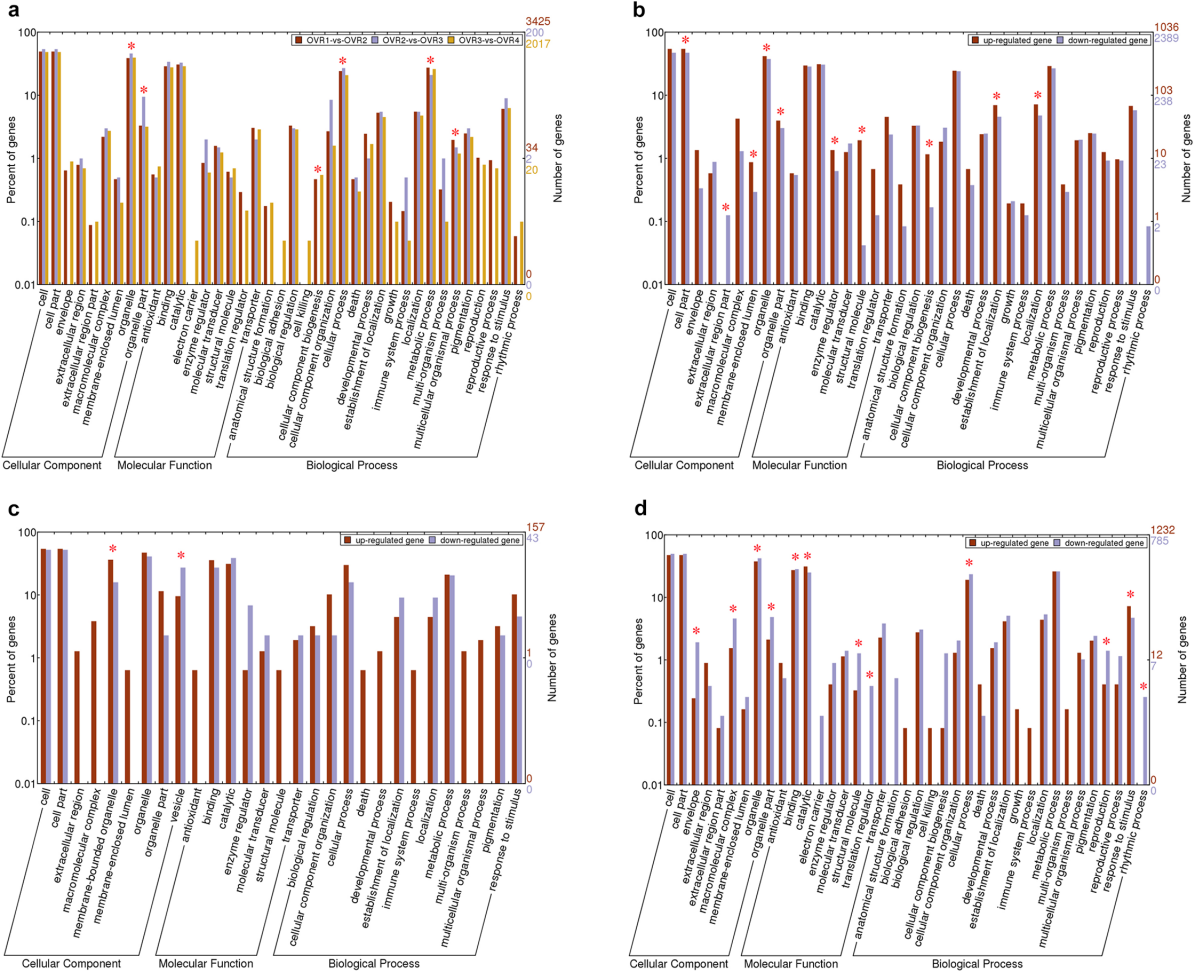
GO classification of DEGs among the adjacent development stages of ovule. **a** GO classification of all DEGs among the three comparisons (OVR1-vs-OVR2, OVR2-vs-OVR3 and OVR3-vs-OVR4). **b** GO classification of up- and down-regulated DEGs between OVR1 and OVR2. **c** GO classification of up- and down-regulated DEGs between OVR2 and OVR3. **d** GO classification of up- and down-regulated DEGs between OVR3 and OVR4.

To determine the functional differences of genes expressed during ovule development and identify genes that may play important roles at each ovule developmental stage, the GO analysis of the DEGs was focused on the adjacent ovule developmental stages (OVR1-vs-OVR2, OVR2-vs-OVR3 and OVR3-vs-OVR4). Using Web Gene Ontology Annotation Plot (WEGO), we categorized the DEGs between OVR1 and OVR2, OVR2 and OVR3, OVR3 and OVR4, and 12, 23 and 9 secondary GO terms were found in cellular component, molecular function and biological process categories, respectively (Fig 6A). The terms metabolic process (GO: 0008152) and cellular process (GO: 0009987) were dominant in the biological process category, demonstrating the probable metabolism and cell activities (such as cell division and differentiation) in ovule during development. In the molecular function category, the terms catalytic (GO: 0003284) and binding (GO: 0001882) were dominant categories. In the cellular components category, cell (GO: 0005623) and organelle part (GO: 0044422) were highly represented (Fig 6A). GO analyses of up-regulated and down-regulated DEGs in the three comparisons were shown in Fig 6B–6D. As observed in Fig 6B, analysis of GO terms in the DEGs of OVR1-vs-OVR2 showed GO terms exhibited statistically significant differences including cell part (GO: 0044464), extracellular region part (GO: 0044421), membrane-enclosed lumen (GO: 0031974), enzyme regulator (GO: 0030234), structural molecule (GO: 0005198), cellular component biogenesis (GO: 0044085), establishment of localization (GO: 0051234), localization (GO: 0051179), organelle (GO: 0043227) and organelle part (GO: 0044422). In Fig 6C, only two terms, membrane-bounded organelle (GO: 0043227) and vesicle (GO: 0031982), showed statistically significant differences. As shown in Fig 6D, a total of 12 GO terms exhibited statistically significant differences in OVR3-vs-OVR4: envelope (GO: 0031075), macromolecular complex (GO: 0032991), organelle (GO: 0043226), organelle part (GO: 0044422), binding (GO: 0005488), catalytic (GO: 0003824), structural molecule (GO:0005198), translation regulator (GO: 0045182), cellular process (GO: 0009987), reproduction (GO: 0000003), response to stimulus (GO: 0050896) and rhythmic process (GO: 0048511).

The results of GO annotations indicated that the DEGs of the ovule developmental process encode diverse structural, regulatory and metabolic proteins, which may participate in various biological processes during the ovule development. The GO annotation of DEGs may provide more insight into the development of the ovule functionally.

### KEGG pathway analysis of DEGs in ovules at four developmental stages

KEGG is a knowledge base for systematic functional analysis of the genes that connects genomic information with higher functional information by computerizing current knowledge on cellular processes and standardizing gene annotations and consists of three databases, GENES, PATHWAY and LIGAND [26]. Genomic information (collection of gene catalogs for all completely sequenced genomes and several partial genomes) is stored in the GENES database; the higher functional information (graphical representation of cell processes, such as metabolism or signal transport) is stored in the PATHWAY database; the LIGAND database stores information regarding chemical compounds, enzyme molecules and enzymatic reactions. To conduct the pathway assignment and functional classification of DEGs during the ovule development, a KEGG analysis was performed.

By mapping to the reference canonical pathways, all DEGs between ovule developmental stages (OVR1-vs-OVR2, OVR2-vs-OVR3 and OVR3-vs-OVR4) were assigned to 125 pathways (S1 Table). The number of pathways significantly enriched with DEGs (*Q* value ≤0.05) in the three comparisons (OVR1-vs-OVR2, OVR2-vs-OVR3 and OVR3-vs-OVR4) was 14, 3 and 4, respectively. In OVR1-vs-OVR2, plant hormone signal transduction, plant-pathogen interaction, starch and sucrose metabolism as well as amino sugar and nucleotide sugar metabolism were significantly enriched pathways. In OVR2-vs-OVR3, the genes were significantly enriched in the pathways of pentose and glucuronate interconversion, plant hormone signal transduction, starch and source metabolism. In OVR3-vs-OVR4, plant hormone signal transduction, starch and sucrose metabolism, amino sugar and nucleotide sugar metabolism and plant-pathogen interaction were significantly enriched by DEGs.

**Fig 7:**
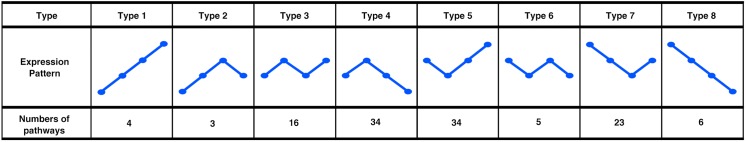
An overview of eight types of expression patterns of the genes from different pathways.

Based on the total RPKM value of the pathway genes at different developmental stages, we identified eight expression types among all pathways (Fig 7 and S1 Table). The genes in the anthocyanin biosynthesis, glycolysis/gluconeogenesis, ether lipid metabolism and histidine metabolism pathways were classified as Type 1, showing up-regulation throughout all four developmental stages. Type 2 contained genes in zeatin biosynthesis, non-homologous end-joining and photosynthesis-antenna protein pathways; gene expression in Type 2 was up-regulated from OVR1 to OVR3 and down-regulated from OVR3 to OVR4. The genes in 16 pathways were classified as Type 3, which were up-regulated from OVR1 to OVR2 and down-regulated from OVR2 to OVR3 and then up-regulated from OVR3 to OVR4. Type 4 contained the genes in 34 pathways, and they were up-regulated from OVR1 to OVR2 and down-regulated from OVR2 to OVR4. The expression of genes in amino sugar metabolism, nucleotide sugar metabolism and base extension repair pathways and the other 32 pathways were classified as Type 5, and they were down-regulated from OVR1 to OVR2 and up-regulated from OVR2 to OVR4. Type 6 contained the genes from five pathways with the expression down-regulated from OVR1 to OVR2, up-regulated from OVR2 to OVR3 and finally down-regulated from OVR3 to OVR4. Type 7 comprised genes from 23 pathways with down-regulated from OVR1 to OVR3 and up-regulated from OVR3 to OVR4. The genes of six pathways were classified as Type 8, and they were down-regulated throughout the entire ovule developmental process.

A summary of the KEGG analysis was shown in Table 3 and S1 Table. The genes in the pathways associated with regulation and metabolism showed significant expression change during ovule development, suggesting the potential roles of these genes in the developmental process of ovule.

**Table 3:** The summary of KEGG pathways with the significantly change of gene expression in ovule development.

	Top10 of expression (OVR+OVR2+OVR3+OVR4)^a^	Top 10 of up-regulated pathways (OVR4–OVR1)^b^	Top 10 of down-regulated pathways (OVR4–OVR1)^c^
1	Metabolic pathways (25,354.6)	Ribosome (5808.3)	ABC transporters (-3,946.2)
2	Ribosome (24,867.4)	Ribosome biogenesis in eukaryotes (995.7)	Plant hormone signal transduction (-3,800.8)
3	Biosynthesis of secondary metabolites (13,954.3)	RNA transport (573.9)	Metabolic pathways (-3,016.8)
4	Plant hormone signal transduction (12,830.6)	Protein processing in endoplasmic reticulum (552.8)	Plant-pathogen interaction (-2,933.6)
5	ABC transporters (10,612.1)	Pantothenate and CoA biosynthesis (497.5)	Biosynthesis of secondary metabolites (-1,319.7)
6	Protein processing in endoplasmic reticulum (8,070.7)	Valine, leucine and isoleucine biosynthesis (475.4)	Starch and sucrose metabolism (-905.6)
7	Plant-pathogen interaction (7,693.5)	Oxidative phosphorylation (381.1)	Phenylpropanoid biosynthesis (-785.4)
8	Ribosome biogenesis in eukaryotes (4,147.1)	Ascorbate and aldarate metabolism (319.0)	SNARE interactions in vesicular transport (-706.1)
9	RNA transport (3,999.3)	Biosynthesis of unsaturated fatty acids (266.3)	Glutathione metabolism (-703.4)
10	Starch and sucrose metabolism (3,995.8)	Glyoxylate and dicarboxylate metabolism (187.4)	Phagosome (-624.8)

**a** represents the total RPKM value of the genes identified in one pathway of four developmental stages.

**b** and **c** represent the total RPKM value of the genes indentified in the pathway in OVR4 minus that in OVR1.

**Fig 8:**
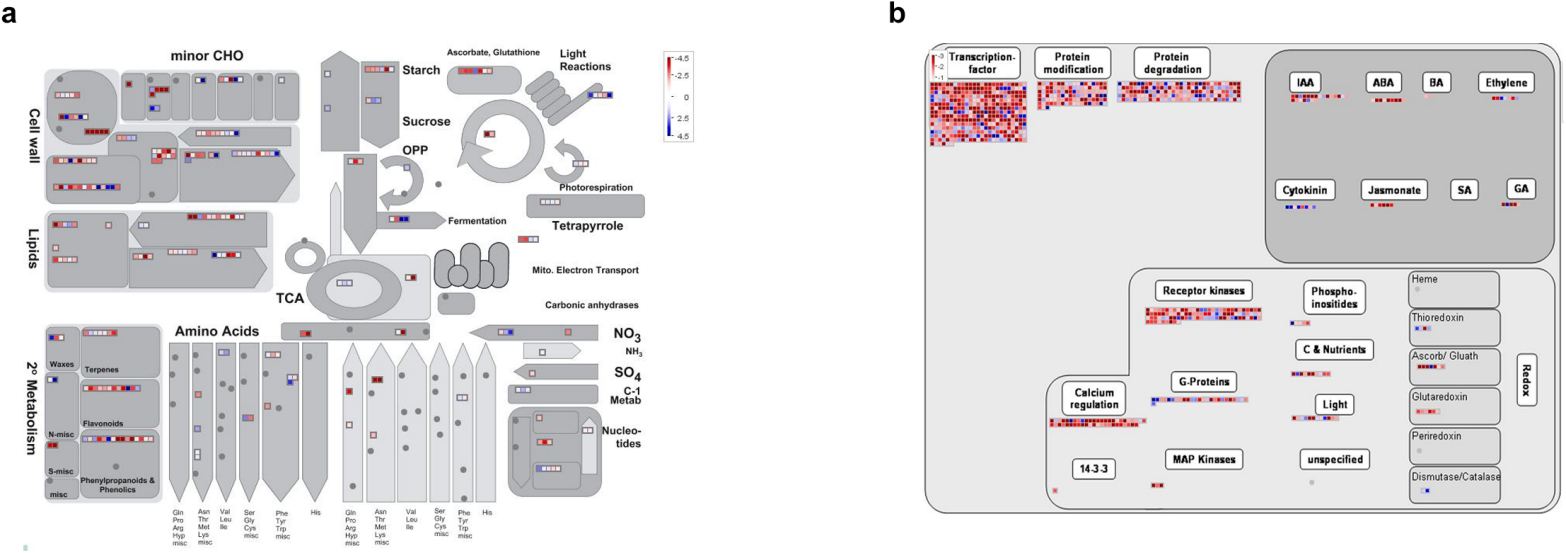
MapMan overview of cellular metabolism (a) and regulation (b)in OVR1-vs-OVR4.Each square represented a gene, and the color key represented the value of RPKM normalized log_2_. Red meant down-regulation of the gene and the blue color meant up-regulation.

The MapMan package (http://MapMan.gabipd.org) was used for a more comprehensive analysis of gene expression in various pathways allowing for more thorough visualization of the pathways involved in ovule development. MapMan uses input from several experts to curate specific biological processes using information from the TIGR database (http://compbio.dfci.harvard.edu/tgi/). According to the gene classification in 35 major pathways and 211 branch pathways, MapMan was used to visualize the pathways during the rice ovule development. To investigate the overall gene expression in terms of cellular metabolism and regulation pathways throughout the entire developmental process of the ovule, we focused on the DEGs between OVR1 and OVR4 (Fig 8). The figure was customized to depict the biological processes of interest and then log_2_ RPKM-normalized expression counts were displayed as pictorial diagrams. As shown in Fig 8, the expression of genes related to transcription factors, protein modification, protein degeneration, receptor kinases, calcium regulation, jasmonate and GA were down-regulated, while genes related to the biosynthesis and transport of cytokinins were up-regulated throughout the developmental process. These visual annotations provided valuable information for understanding the pathways involved in ovule development and suggested that potential genes act cooperatively.

### Expression pattern analysis of DEGs in ovules at four developmental stages using hierarchical clustering

**Fig 9:**
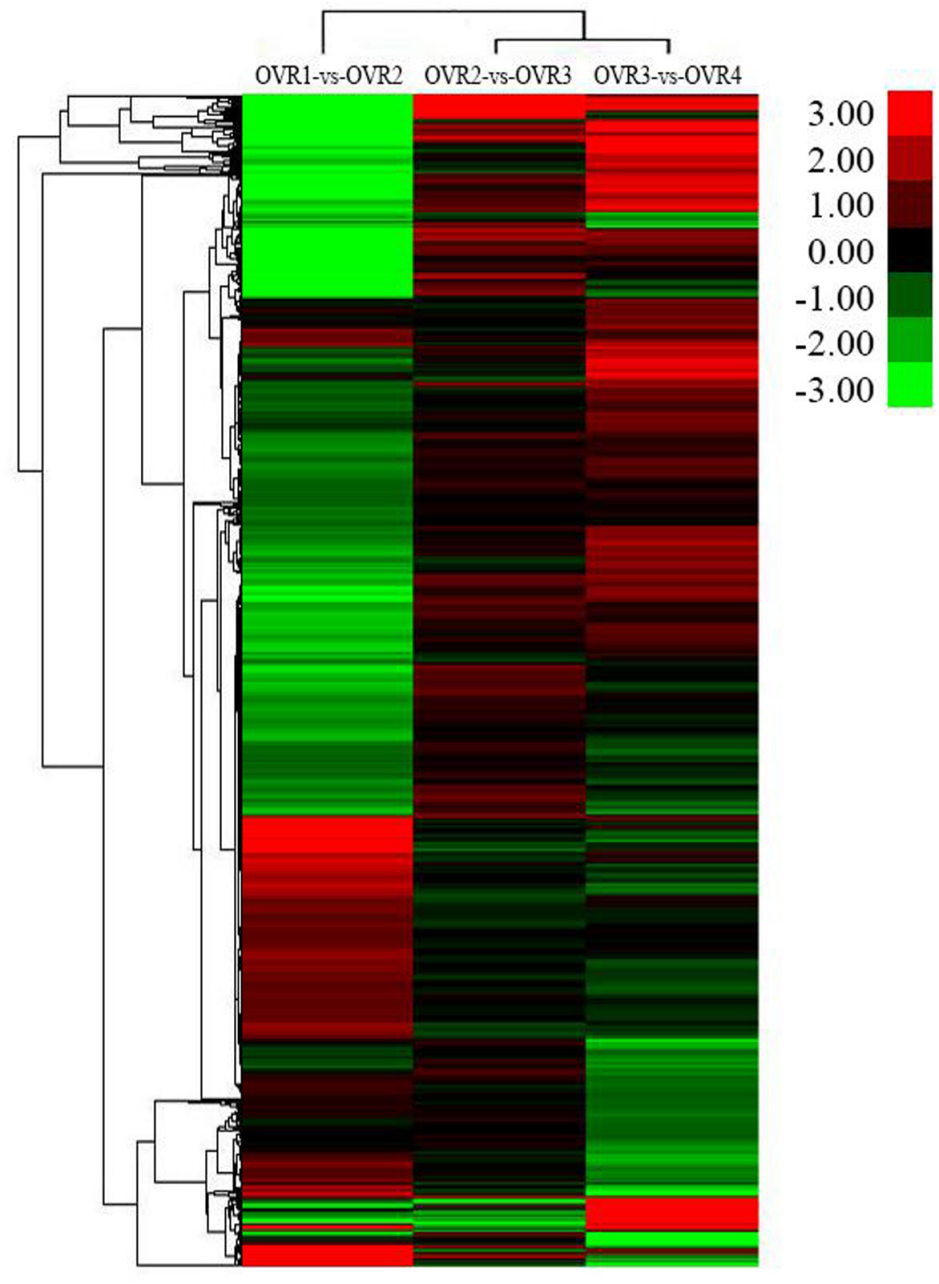
Hierarchical cluster analysis of gene expression based on log ratio RPKM data. The color key represented RPKM normalized log_2_transformed counts. The green color represented lower expression, and the red color represented higher expression. Each column represented an experimental condition, each row represented a gene.

Genes with similar expression patterns usually indicate functional correlations. Cluster analysis of gene expression patterns was performed using the cluster software and JAVA tree view software. Using the Pearson correlation as the distance metric, a cluster analysis of gene expression patterns was performed (Fig 9 and S2 Table). Expression disparities were visualized as different colors; red indicated up-regulation and green down-regulation. The hierarchical clustering can provide an overall understanding of DEGs. A large proportion of genes were down-regulated in OVR1-vs-OVR2, up-regulated in OVR2-vs-OVR3; and the number of up-regulated genes was approximately identical to the number down-regulated in OVR3-vs-OVR4.

Based on the expression level of genes at different developmental stages, 4,405 DEGs from three comparisons (OVR1-vs-OVR2, OVR2-vs-OVR3 and OVR3-vs-OVR4) were classified into eight clusters according to their expression patterns. Cluster 1 consisted of 107 genes whose expression was up-regulated from OVR1 to OVR4. Cluster 2 had 370 genes that were up-regulated from OVR1 to OVR3 and down-regulated from OVR3 to OVR4. Cluster 3 contained 281 genes whose expression was up-regulated from OVR1 to OVR2, down-regulated from OVR2 to OVR3 and then up-regulated to OVR4. Genes that were up-regulated from OVR1 to OVR2 and down-regulated from OVR2 to OVR4 were defined as Cluster 4, which contained 772 genes. Cluster 5 comprised the largest number of genes (1,460 genes), and the expression of which was down-regulated from OVR1 to OVR2 and up-regulated to OVR4. Cluster 6 contained 752 genes that were down-regulated from OVR1 to OVR2, up-regulated from OVR2 to OVR3 and down-regulated from OVR3 to OVR4. Genes that were down-regulated from OVR1 to OVR3 and up-regulated to OVR4 were classified as Cluster 7, which contained 503 genes. Cluster 8 consisted of 160 genes with down-regulated expression throughout the four developmental stages.

### Transcription factor (TF) gene expression analysis during the ovule developmental stages

Transcriptional regulation of gene expression depends on the recognition of promoter elements by TFs. Thus, TFs play an important role in regulating a series of events, such as growth, development and response to stress. To identify the TF genes expressed during the development of rice ovule and determine those crucial for ovule development, we blasted our data to the rice TF database (http://drft.cbi.pku.edu.cn/). In total, 1,431 putative TF genes were identified from the 28,728 genes detected in the ovule and they were classified into 56 gene families. According to the RPKM, 394 genes of the 1,431 identified putative TF genes showed significantly differential expression, accounting for 27.5% of all TF genes (S3 Table).

According to the overall changes in total RPKM value of ovules in the four developmental stages for each family, genes encoding the 56 transcription families were classified into eight different expression patterns (S4 Table).

The overall gene expression pattern encoding the ARF family was classified as pattern 1, which showed an up-regulated trend throughout the entire developmental process. The C2H2 family and five other families were classified as expression pattern 2, which were up-regulated from OVR1 to OVR3 and down-regulated from OVR3 to OVR4. The Whirly family was classified as pattern 3, which was up-regulated from OVR1 to OVR2, down-regulated from OVR2 to OVR3 and up-regulated from OVR3 to OVR4. The genes encoding the VOZ family and two other families were classified as pattern 4, which were up-regulated from OVR1 to OVR2 and down-regulated from OVR2 to OVR4. The BES1 family and the other 18 families were classified as pattern 5 and exhibited down-regulation from OVR1 to OVR2 and up-regulation from OVR2 to OVR4. Pattern 6 had the largest number of families, which included those in AP2 and 19 other TF families that showed down-regulation from OVR1 to OVR2, up-regulation from OVR2 to OVR3, followed again by down-regulation. The EIL family was classified as pattern 7, which was down-regulated from OVR1 to OVR3 and then up-regulated at OVR4. The BBR-BPC family and four other families were classified as pattern 8, expression of which was down-regulated throughout ovule development. More detailed information regarding the TF gene expressions involved in the ovule development was shown in S4 Table.

### Expression of plant hormone-related genes in rice ovule

Plant hormones regulate many aspects of plant life. As the messengers of cross-talk between sporophyte and female gametophyte in the ovule, plant hormones, especially auxin and cytokinin, are important during the regulation of developmental processes [27, 28]. Thus, we focused on these two important plant hormone-related genes, which encode proteins participating in the biosynthesis, transport, perception and response of these hormones. These genes may play important roles in the cross-talk between sporophyte and female gametophyte. In total, 135 auxin-related genes and 43 cytokinin-related genes were identified during ovule development.

Auxin regulates cell division, elongation and differentiation in plants [29]. In *Arabidopsis*, manipulation of auxin responses or synthesis induces switching of gametic and nongametic cell identities [30]. Based on the transcriptome data, 27 of the 135 identified auxin-related genes were related to the efflux of auxin; and the majority of the others were related to the auxin response. In the OVR1-vs-OVR2 comparison, 29 auxin genes were significantly differently expressed and 18 genes were down-regulated. In the OVR2-vs-OVR3 comparison, only four up-regulated auxin-related DEGs were found. Compared with OVR3, 16 auxin genes were significantly differently expressed and 9 genes were up-regulated in OVR4. The results indicate that most genes related to the biosynthesis of auxin were expressed at the initial developmental stage of the ovule and then showed down-regulation. The genes related to the transport of auxin showed an expression pattern similar to that of the auxin biosynthesis-related genes, while the genes related to the response regulator showed relatively constant expression. The expression of these genes at various developmental stages suggests that auxin may act as a signal molecule between sporophytic ovule tissue and the female gametophyte in the developing ovule.

Cytokinin is another plant hormone signal involved in cross-talk between sporophyte and female gametophyte during the ovule development [31]. Among the 43 identified cytokinin-related genes, 33 genes were related to the modification of cytokinin, which may regulate its activity. In the OVR1-vs-OVR2 comparison, one up-regulated DEG and four down-regulated DEGs were found. Compared with OVR2, no cytokinin-related gene exhibited differential expression in OVR3. Two cytokinin-related DEGs were identified in the OVR3-vs-OVR4 comparison; one was up-regulated and another one was down-regulated. According to the statistical data, the genes related to the biosynthesis of cytokinin exhibited higher expression in OVR1 and OVR2, and the genes related to cytokinin transport showed a similar expression pattern.

Consequently, genes related to the response to these two plant hormones exhibited constitutive expression during ovule development. However, the DEGs were related mainly to the biosynthesis and transport of plant hormones. Although expression level diversities existed between the two plant hormone-related DEGs, the expression levels of transport-related and biosynthesis-related genes were closely correlated, possibly reflecting the different demands of these two plant hormones during specific developmental stages and pointing out the potential mechanisms of plant hormone regulation during the ovule development.

### Validation of RNA-seq data using quantitative real-time PCR (qRT-PCR)

To validate the obtained RNA-seq data, qRT-PCR was performed. A total of 19 genes were selected stochastically. Gene-specific primers for qRT-PCR were designed using the Primer 5.0 software (S5 Table). The validation results were shown in S3 Fig. The expression patterns of the selected genes determined by qRT-PCR were consistent with the RNA-seq data, indicating the reliability and accuracy of the RNA-seq data.

## Discussion

### Establishment of an overall transcriptome analysis for rice ovule development

The transcriptome data presented here establish a genome-wide transcriptional landscape for studies of developmental and molecular aspects of ovule development in rice. The transcriptome data obtained using Illumina RNA-seq in our research was larger in comparison to a recent microarray-based analysis of rice ovule transcriptome [20] and more genes were detected at each stage of ovule development. Several similarities and differences exist between our and the previous studies. These differences may be caused by the use of two different technologies (RNA-seq or microarray), which may affect the results. Second, two different subspecies of rice (*Oryza sativa* ssp. *indica* or ssp. *japonica*) were used as the experimental plant material, respectively. Third, we focused not only on the ovule gene expression before fertilization but also the ovule gene expression at two days after fertilization. Additionally, DEGs between developmental stages were identified and analyzed; thus genes potentially involved in ovule development were identified. DEGs between developmental stages were also classified into several pathways to facilitate identification of those involved in physiological activities.

Previous research demonstrated that some genes were stage-specifically or cell-type-specifically expressed while other genes had relatively constant expression level in the ovule [32]. In this study, we also identified genes expressed at only one developmental stage, and 1,230, 401, 485 and 532 genes were found to be expressed in ovules at the four developmental stages, respectively. The *OsDMC1* gene (LOC_Os11g04954) is regarded as essential for meiosis and plays an important role in homologous pairing [33]. We identified this gene in the ovule transcriptome and its expression level was highest in OVR1, providing new genetic evidence for its importance in meiosis. The *MEL1* gene regulates the proper modification of meiotic chromosomes [34], and it was identified in OVR1. *In situ MEL1* was expressed at a high level in the megaspore mother cell in the developing ovule [34]. According to the ovule transcriptome results, *MEL1* expression was significantly higher in OVR1, suggesting it may be a marker of OVR1. However, few genes have been reported as markers of the OVR2stage. From the rice ovule transcriptome data, we identified 401 genes that were expressed only in OVR2; these may include candidate marker genes of this stage. The *BLH1* gene identified in *Arabidopsis* could affect cell fates in the mature embryo sac [23]. Its homolog was identified in the rice ovule transcriptome, and was highly expressed in OVR3. Genes expressed in OVR4 were the subjects of the last part of the discussion.

Approximately 27,000 genes have been identified in rice egg cells [35], and 28,728 genes were detected in rice ovule in this study, indicating that a greater number of genes were expressed in the whole ovule than in the egg cell, probably because the egg cell accounts for a very small proportion of the ovule. Although many genes in the whole ovule and embryo sac showed similar expression levels [36], the expression levels of some genes actually differed and displayed different enrichment of GO items. Genes were enriched in the GO terms protein binding, nucleus and mitochondrion in the egg cell, and enriched in terms membrane, transporter activity and endoplasmic reticulum in the synergid. In contrast, genes were enriched in the GO terms cell part, metabolic process, cellular component organization and organelle in the ovule. The different enrichment of GO terms among cell types or tissues is suggestive of their various biological functions during the developmental process.

In addition, genes involved in starch and sucrose metabolism, ABC transporters, and amino sugar and nucleotide sugar metabolism pathways showed specific enrichment, likely with the purpose of guaranteeing the energy and material necessary for ovule and embryo sac development. In particular, 26 genes were identified in the starch and sucrose biosynthesis pathways and 27 genes were identified in the pentose and glucuronate conversion pathways. Based on KEGG analysis, genes involved in the pentose and glucuronate interconversion pathways and starch and sucrose pathways were significantly up-regulated from OVR2 to OVR4 (S1 Table), indicating their potential involvement in utilizing nutrition and increasing energy during the various stages of ovule development, likely not only for sporophyte tissue but also for female gametophyte (embryo sac) development.

Overall, the DEGs from developmental stages may play various roles in ovule development and the identification of several key genes will enable further functional studies.

### Genes expressed in ovule sporophyte tissue may play important roles in its cross-talk with female gametophyte

In rice ovule, the female gametophyte is embedded and sustained by the sporophytic tissue. Previous reports showed cross-talk communication between the female gametophyte and the sporophyte ovule tissue [37]. This communication is particularly important for megasporogenesis regulation and female gametophyte development. Recent studies have reported that several plant hormones (especially auxin and cytokinin) and peptides act as signal molecules in this cross-talk [16, 27, 29]. Thus, we focused on the hormone-related genes that may play important roles in the cross-talk between the sporophyte and female gametophyte during ovule development.

Auxin gradients have been reported to control female gametophyte cell identity [29]. In the *Arabidopsis* ovule, the import and local biosynthesis of auxin are required for cell expansion, mitotic divisions and cell specification during the development of female gametophyte, and auxin distribution changes together with embryo sac development [38]. When the functional megaspore is visible, a strong auxin signal can be detected in the sporophytic ovule tissue, and the auxin signal peaked at the micropylar end of the embryo sac when the third mitotic division in the embryo sac was completed [29]. This observation clarified the direction of auxin transduction in the ovule. In our research, 135 auxin-related genes were identified. In particular, genes from two auxin-related gene families (OsPIN gene family and OsYUCCA gene family) were the focus of our interests. The OsPIN gene family is the homologue of PIN family in *Arabidopsis*. *PIN1*, which encodes an auxin efflux carrier protein, is expressed at only one side of the nucellar cell [27]. In our results, four members of the OsPIN gene family were found: *OsPIN1a* (LOC_Os06g12610), *OsPIN1b* (LOC_Os02g50960), *OsPIN1c* (LOC_Os11g04190) and *OsPIN1d* (LOC_Os12g04000). Expression of all of these four genes was higher in OVR1 and was down-regulated significantly in OVR2. The expression of these four genes suggests that auxin transport in the ovule may be strictly regulated, and this regulation may be important in ovule development. The *YUCCA* genes, encoding a putative flavin monooxygenase (the key enzyme in auxin biosynthesis), are expressed in the micropylar region of the nucellus [39]. In this study, two members of the OsYUCCA gene family were found: *OsYUC3* and *OsYUC9*, both of which exhibited higher expression levels in OVR1. OVR1 represented the ovule containing the megaspore mother cell during meiosis; thus, the higher expression of these types of auxin-related genes suggests that these two auxin-related genes may have the same function as the *PIN* and *YUCCA* genes in sporophytic ovule tissue in *Arabidopsis*, and they probably participate in the cross-talk between sporophyte ovule tissue and female gametophyte.

The plant hormone cytokinin promotes cell proliferation and differentiation [40]. A series of genes are involved in the sporophyte-gametophyte cross-talk induced by cytokinin; e.g., *IPT* (genes encoding isopentenyl transferase, the key enzyme in cytokinin biosynthesis [41]), *AHK2*, *AHK3*, *AHK4* (encoding receptor histidine protein kinases [42]), and *RR* (encoding a response regulator, promoting rapid induction of cytokinin-related target genes [43]). Specifically, *AtIPT1* (the homologous *IPT* gene in *Arabidopsis*) is strongly expressed in the chalazal part of the ovule, indicating that the source of the cytokinins perceived by the gametophyte is located in the sporophyte ovule tissue [27]. In our study, *OsIPT1* and *OsIPT3* were found to be highly expressed in OVR1. This suggests that the biosynthesis of this plant hormone begin at the initial stage of ovule development. Regarding hormone regulation, a proper balance of cytokinins is important for ovule organogenesis. ARR-A type genes (*ARR3*, *ARR4*, *ARR5*, *ARR6*, *ARR7*, *ARR8*, *ARR9*, *ARR15*, *ARR16*and *ARR17*) play roles as negative regulators of the cytokinin response [44]. In *Arabidopsis*, mutant *arr7* and *arr15* showed megagametophyte defects [45]. In *coatlique* and *sporocyteless* mutants (both of which lack a functional embryo sac), A-type ARR genes were found to be overexpressed compared with the wild-type, indicating negative regulation of A-type ARR genes in ovule development. We identified seven homologous genes of A-type ARR in the rice ovule transcriptome data: *OsRR1*, *OsRR2*, *OsRR3*, *OsRR4*, *OsRR5*, *OsRR6* and *OsRR10*. Of these, *OsRR2*, *OsRR3* and *OsRR4* expression peaked in OVR2, while *OsRR1*, *OsRR6* and *OsRR10* expression was lowest in OVR2. *OsRR3* was identified only in OVR3. The expression of these genes possibly proves that the negative regulation of sporophyte-gametophyte cross-talk may also exist during the ovule development, which may play important roles in guaranteeing the cross-talk functions in orderly manner combined with positive regulation. Moreover, OsPUP family genes have also been identified. This family encodes purine permeases, transporters of cytokinin. In this work, nine members of the OsPUP family were identified: *OsPUP2*, *OsPUP3*, *OsPUP4*, *OsPUP5*, *OsPUP6*, *OsPUP7*, *OsPUP8*, *OsPUP10* and *OsPUP11*. *OsPUP7* was identified in only the later two stages of ovule development before fertilization, suggesting that *OsPUP7* may have a special role in ovule development. In a study of *OsPUP7* [46], T-DNA insertion caused multiple phenotypic changes, including increased plant height and delayed flowering, but the detailed role of *OsPUP7* in ovule development has not been investigated. The above data indicate the complex and highly ordered plant-hormone-mediated cross-talk between the sporophytic ovule tissue and the female gametophyte. These cross-talk signal molecules may be essential to the process of female gametophyte formation.

### The effects of fertilization on gene expression in the rice ovule

Fertilization is a key process during rice ovule development. Upon double fertilization, a sperm cell fuses with an egg cell and the resultant zygote develops into an embryo, while the central cell fuses with a second sperm cell and develops into the endosperm [47]. After fertilization, three major components of the ovule (sporophytic ovule tissue, primary endosperm nucleus and zygote) undergo different cell fates [48]. Two days after fertilization, the zygote undergoes cell division to form a globular embryo with no apparent morphological differentiation. Synchronously, the primary endosperm nucleus undergoes the first division and forms the two free nuclei of the endosperm, which then undergo further rounds of division. The sporophytic ovule tissue provides nutritive support for the development of the embryo and endosperm by undergoing PCD-triggered degradation after fertilization [5].

PCD during the sporophytic ovule tissue degeneration after fertilization is critical for the proper development of the embryo in flowering plants. In rice, PCD in the tapetum is governed by the API5-AIP1/2-CP1 pathway [5]. *OsAPI5* (APOPTOSIS INHIBITOR5), which encodes a putative homolog of the animal API5 protein, is a critical positive regulator of PCD. The knockdown of *OsAPI5* resulted in delayed tapetum degeneration [5]. AIP1 (API5-INTERACTING PROTEIN1) and AIP2 are DEAD-BOX ATP-dependent RNA helicases. The protein encoded by *CP1* acts as an executor of the PCD process, resulting in the initiation of cell degeneration in *Arabidopsis* [49]. *OsCP1*, the homolog of *CP1* in rice, plays an important role in the tapetum PCD process [50, 51]. In the rice ovule transcriptome, the *OsAIP1*/*2* expression level increased from OVR3 to OVR4 and *OsCP1* was up-regulated in OVR3-vs-OVR4, indicating that the PCD process of ovule sporophytic tissue after fertilization may be mediated by the same mechanism as the tapetum PCD. This up-regulation of related genes may provide information for future studies focusing on the mechanism of the PCD process in ovule sporophytic tissue after fertilization.

Conversely, the free nuclei of endosperm in ovule undergo several rounds of division after fertilization. Several genes participate in endosperm development in *Arabidopsis*, such as *AtTIM9* and *AtTIM10*, which encode translocases of the inner membrane and regulate endosperm free-nuclei division, and dysfunction of *AtTIM9* or *AtTIM10* leads to arrest of endosperm division [52]. The RNA-seq results showed that *TIM9* and *TIM10* were up-regulated in rice ovules at the OVR4 stage, suggesting that these genes may also function to control endosperm free-nuclei division during early endosperm development in the rice ovule.

After fertilization, the developmentally quiescent egg cell in the ovule is transferred to the zygote. The synthetic pathways are activated and several receptor kinase-related genes are up-regulated in the zygote, suggesting that a series of biological processes is also activated following fertilization [53]. Based on our findings, 11,958 genes were up-regulated in OVR3-vs-OVR4, 1,232 of which exhibited significant up-regulation. In the up-regulated DEGs, GO annotation showed enrichment of binding, organelle, cellular process and response to stimulus, and genes involved in cell wall biosynthesis, auxin and ethylene exhibited significant up-regulation from OVR3 to OVR4. These data are in agreement with the activation of zygote division in rice ovule, suggesting a role in pre-embryo development. Wuschel-related homeobox 2 (*WOX2*) is a key fertilization-induced gene that plays an important role in determining cell fate of the ovule after fertilization [54, 55]. In the *Arabidopsis* ovule, *WOX2* was expressed only in the apical cell of the two-celled pro-embryo [56], and is the dominant regulator of apical patterning [57]. The *OsWOX2* was also up-regulated in rice from OVR3 to OVR4, suggesting that this gene may play an important role in the initiation of zygote development in the rice ovule.

Additionally, we were interested in the *MET1* gene, which encodes DNA methyltransferase 1. *MET1*is induced by fertilization and plays an important role in early zygotic development [58]. In *Arabidopsis*, *MET1* expression was suppressed in the egg and central cell at the end of female gametophyte development [59]. After fertilization, *MET1* was expressed to maintain DNA methylation only in the young embryo [60]. Conversely, the loss-of-function mutant *met1* showed abnormal development beginning at initiation of fertilization [61]. The above reports indicate that the induction of *MET1* expression may be important for zygotic development and early embryogenesis in *Arabidopsis*. In our study, expression of *OsMET1* (LOC_Os07g08500), the *MET1* homolog in *Arabidopsis*, was up-regulated from OVR3 to OVR4. This up-regulation may facilitate the effects of *MET1* expression on zygotic development and early embryogenesis in the ovule. Summarily, the expression levels of many genes associated with embryo and endosperm development were increased from OVR3 to OVR4 in the ovule, and further research on the exact function of these genes in rice ovule development after fertilization is needed.

In conclusion, transcriptomic analysis using RNA-seq represented a comprehensive and intensive method of evaluating rice ovule development. Our study produced sufficient and reliable data for the investigation of rice ovule development, and DEGs among the four stages were identified, indicating their potential roles in the developmental process. GO and KEGG analysis showed that genes related to cellular component biogenesis, membrane-bounded organelles and reproductive regulation were highly expressed during the ovule development. Additionally, we showed the important role of the ovule sporophytic tissue in megasporogenesis and development of the female gametophyte by identifying the plant hormone-related genes and nutrition-related genes with altered expression. The transcriptome data will facilitate further studies of the complex regulation of gene expression during rice ovule development and enable elucidation of the molecular mechanisms of female gametophyte development processes in rice, as well as other plants.

## Supporting Information

S1 Fig The DIC observation of four developmental stages of ovule. **A** and **B** showed morphological features inside the ovule in meiosis stage (OVR1). **C** showed morphological features inside the ovule in mitosis stage (OVR2). **D** showed morphological features inside the ovule in mature embryo sac stage (OVR3). **E** showed morphological features inside the ovule in fertilized stage (OVR4). **Mmc**, megaspore mother cell. **Mt**, megaspore tetrad. **Ac**, antipode cells. **Cc**, central cell. **Ec**, egg cell. **S**, synergid. **Em**, embryo.
(TIF)

S2 Fig The sequencing saturation analysis of the five samples. (TIF)

S3 Fig The result of real-time qPCR validation, showing the relative expression of 19 selected genes. The bars represented the standard deviation.
(TIF)

S1 Table An overview of all DEGs between ovule developmental stages (OVR1-vs-OVR2, OVR2-vs-OVR3, OVR3-vs-OVR4) assigned to 125 KEGG pathways. The KEGG functional class, number, RPKM of four stages and expression type were all presented in the table.
(XLS)

S2 Table The list of the differentially expressed genes (DEGs) during ovule development (OVR1-vs-OVR2, OVR2-vs-OVR3, OVR3-vs-OVR4). (XLS)

S3 Table All 56 TF gene families were found in the result. Of the 1431 putative TF genes, the genes which showed significantly differential expression among four stages were marked by *. The TF gene family name, number, RPKM were presented in the table.
(XLS)

S4 Table A summary of TF gene families. The TF gene family name, number, RPKM, expression pattern and expression pattern of TF gene family’s members were presented in the table.
(XLS)

S5 Table Primers used in the analysis of rice ovule development. *OsActin1* (LOC_Os03g50885) was used as internal control to standardize the results.
(XLS)
