# Supplementary figures and images for: Gene Expression Profiles in Rice Developing Ovules Provided Evidence for the Role of Sporophytic Tissue in Female Gametophyte Development
Source: PLoS One. 2015 Oct 27;10(10):e0141613. doi: 10.1371/journal.pone.0141613 (PMC4624635; doi:10.1371/journal.pone.0141613)

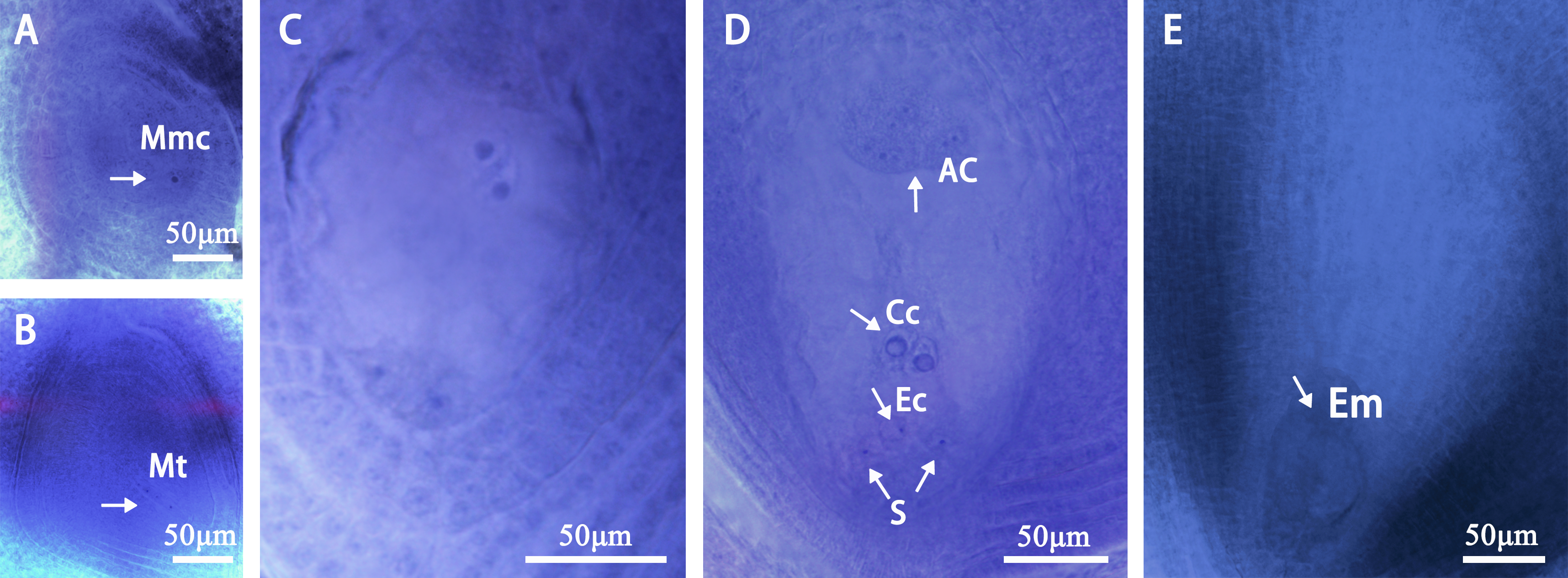

Supplement: S1 Fig — A and B showed morphological features inside the ovule in meiosis stage (OVR1). C showed morphological features inside the ovule in mitosis stage (OVR2). D showed morphological features inside the ovule in mature embryo sac stage (OVR3). E showed morphological features inside the ovule in fertilized stage (OVR4). Mmc, megaspore mother cell. Mt, megaspore tetrad. Ac, antipode cells. Cc, central cell. Ec, egg cell. S, synergid. Em, embryo. (TIF) [file pone.0141613.s001.tif]

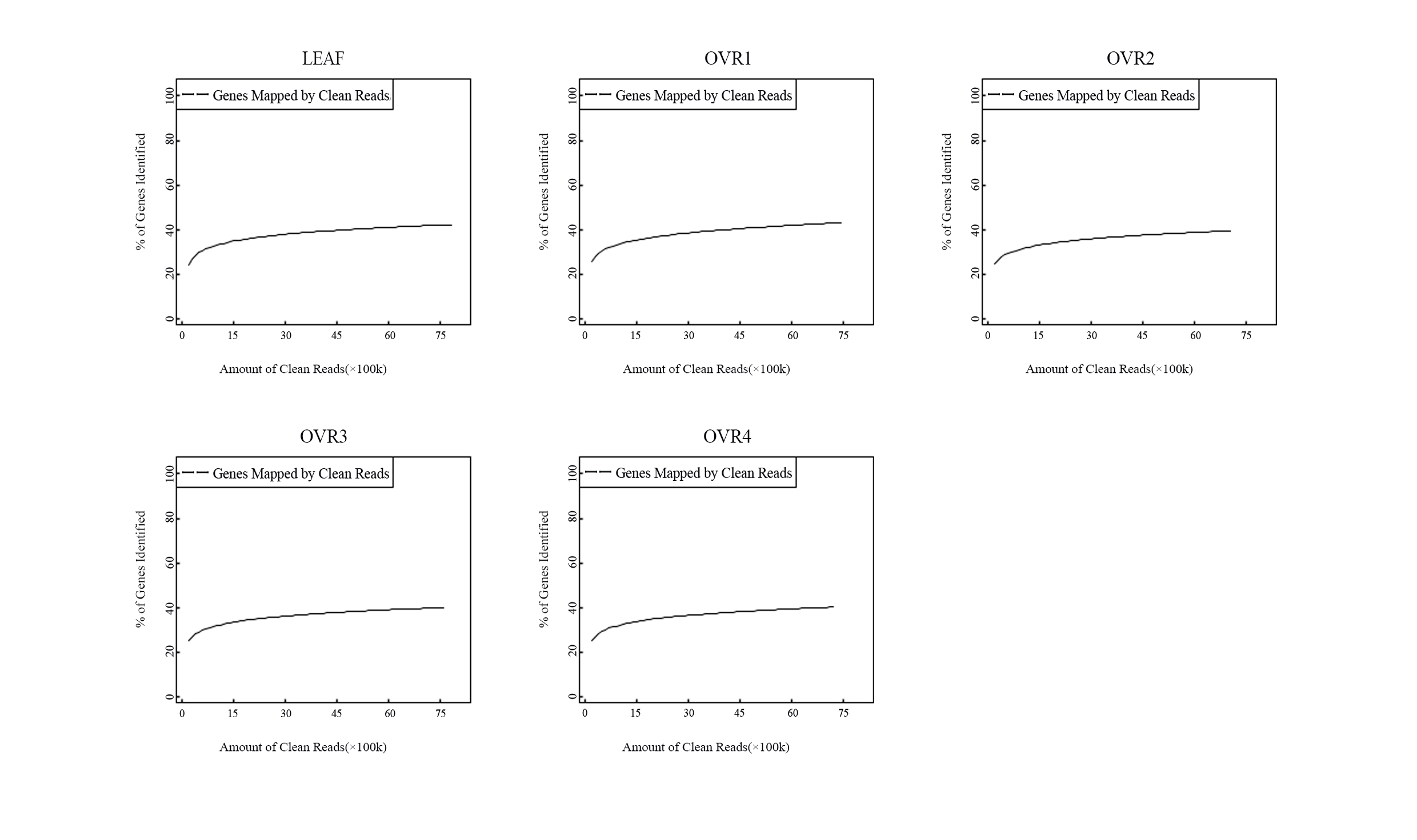

Supplement: S2 Fig — (TIF) [file pone.0141613.s002.tif]

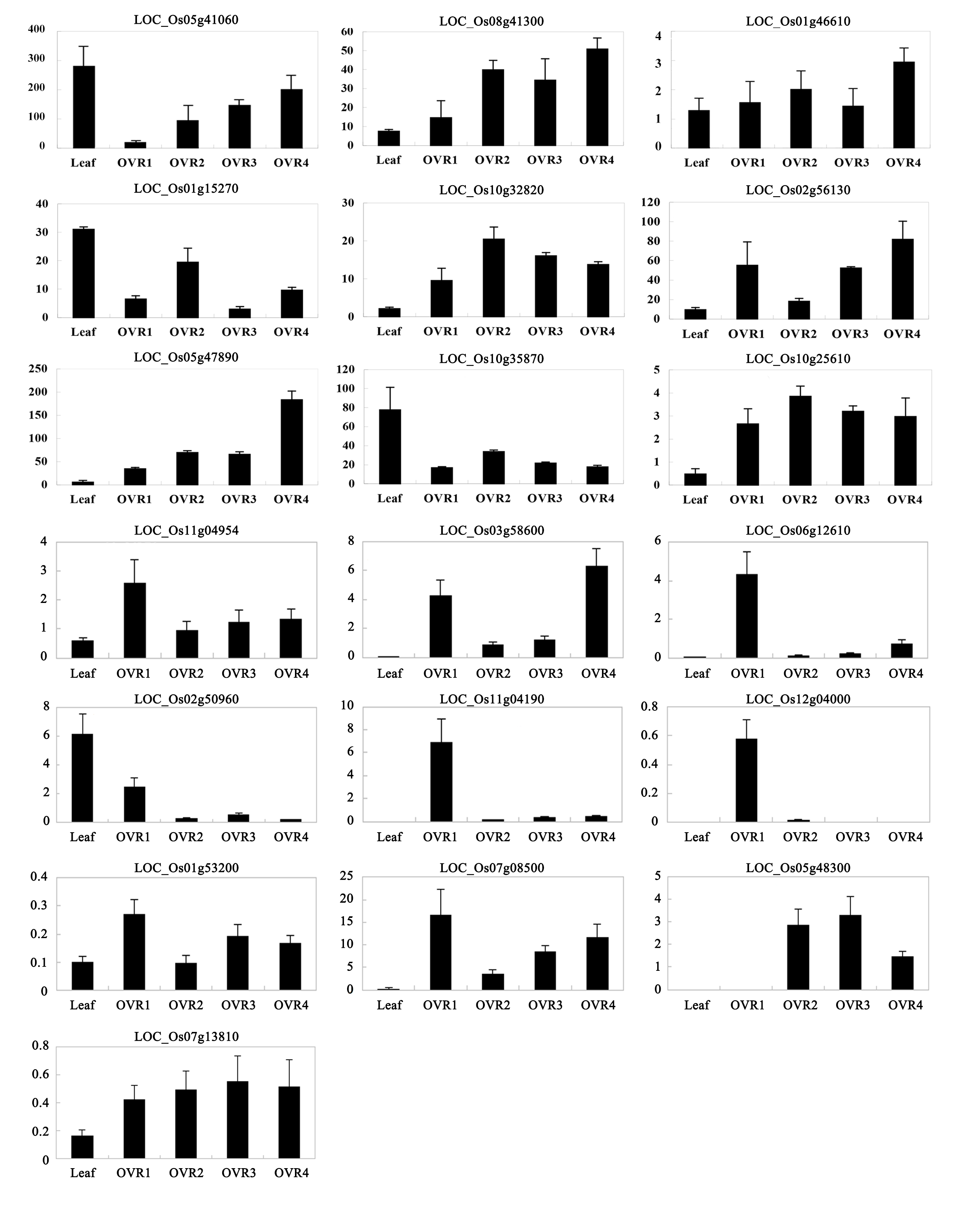

Supplement: S3 Fig — The bars represented the standard deviation. (TIF) [file pone.0141613.s003.tif]
